# A randomized controlled trial of the effect of raloxifene plus cholecalciferol versus cholecalciferol alone on bone mineral density in postmenopausal women with osteopenia

**DOI:** 10.1093/jbmrpl/ziae073

**Published:** 2024-05-30

**Authors:** Sungjae Shin, Namki Hong, Yumie Rhee

**Affiliations:** Division of Endocrinology and Metabolism, Department of Internal Medicine, National Health Insurance Service Ilsan Hospital, Goyang 10444, Republic of Korea; Department of Internal Medicine, Endocrine Research Institute, Yonsei University College of Medicine, Seoul 03722, Republic of Korea; Department of Internal Medicine, Endocrine Research Institute, Yonsei University College of Medicine, Seoul 03722, Republic of Korea; Department of Internal Medicine, Endocrine Research Institute, Yonsei University College of Medicine, Seoul 03722, Republic of Korea

**Keywords:** raloxifene, randomized controlled trial, osteopenia, bone mineral density, postmenopausal women

## Abstract

Raloxifene increases lumbar spine bone mineral density (BMD) and lowers vertebral fracture risk in patients with osteoporosis. However, few prospective clinical trials have studied its efficacy in postmenopausal women with osteopenia. This study investigated the efficacy of raloxifene in postmenopausal women with osteopenia. An investigator-initiated, randomized, open-label, prospective, single-center trial was conducted in 112 postmenopausal women with osteopenia. Osteopenia was defined based on the lowest BMD T-score in the lumbar spine, femoral neck, or total hip (−2.5 < lowest T-score < −1.0). Participants were randomly assigned to receive raloxifene 60 mg/day plus cholecalciferol 800 IU/day (RalD) or cholecalciferol 800 IU/day (VitD) for 48 wk. At baseline, mean age (63.1 ± 6.8 yr) did not differ between the two groups. However, in the RalD group, mean body mass index (BMI) and baseline T-score were lower, while 25-hydroxyvitamin D level was higher. At 48 wk, the RalD group showed a greater increase in lumbar spine BMD (RalD vs. VitD; 2.6% vs. −0.6%, *P* =.005) and attenuated the total hip BMD loss (−0.3% vs. −2.9%, *P* = .003). The effect of raloxifene on the lumbar spine remained significant after adjustment for age, BMI, baseline BMD T-score, and other covariates (adjusted β: +3.05 vs. VitD, *P* =.015). In subgroup analysis, the difference in lumbar spine BMD between the RalD and VitD groups was robust in those with severe osteopenia group (lowest T-score ≤ −2.0). Raloxifene plus cholecalciferol significantly improved lumbar spine BMD and attenuated total hip BMD loss compared with cholecalciferol alone, with a more robust effect in severe osteopenia.

*Clinical trial registration*: The trial was registered with ClinicalTrials.gov (NCT05386784).

## Graphical Abstract

**Graphical Abstract:**
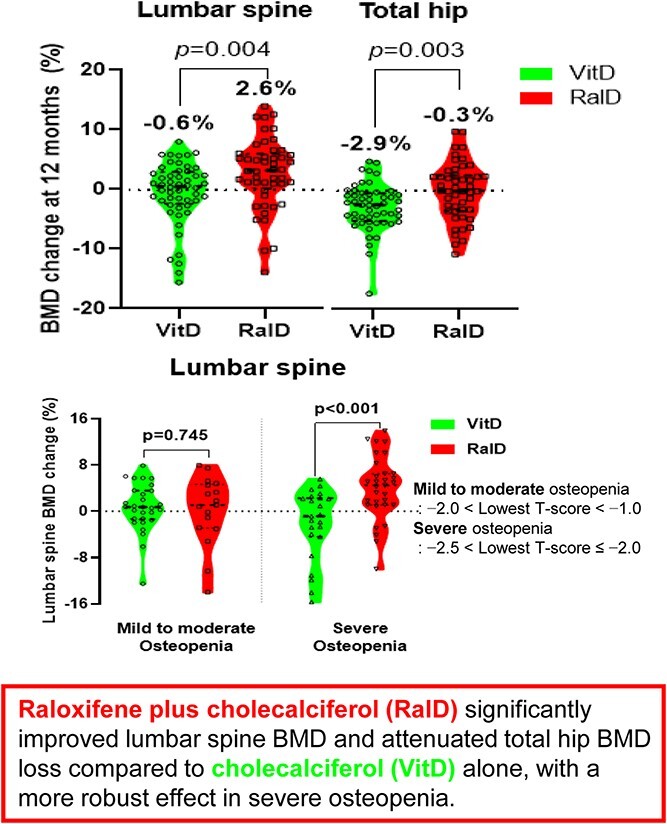

## Introduction

Osteopenia is a clinical term referring to a condition in which bone mineral density (BMD) falls below normal levels but does not reach the diagnostic criteria for osteoporosis.^1^ Current guidelines generally state that diagnostic criteria are primarily applicable for osteoporosis, while osteopenia (or low bone mass) should be categorized not as a disease but rather as an epidemiological description.^2^^,^^3^ While pharmacological treatment is widely accepted for patients with common osteoporotic fractures, treating those at high risk of fracture due to osteopenia is controversial.^4^ However, in the prospective population-based cohort Rotterdam Study, 44% of nonvertebral fractures occurred in patients with osteoporosis and a similar proportion (43%) in those with osteopenia.^5^ In the National Osteoporosis Risk Assessment study, 82% of postmenopausal women with fractures had T-score higher than −2.5.^6^ Given that the number of osteoporotic fractures has been steadily increasing since 2008 in Korea, there is a need for more proactive measures to prevent fractures in postmenopausal women with osteopenia.^7^

The effectiveness of pharmacological interventions in mitigating fracture risk in postmenopausal women with osteopenia is still under investigation. The Reid et al. study,^8^ a landmark trial in osteopenia, showed that zoledronate increased BMD and reduced fracture incidence in postmenopausal women aged 65 years or older with femoral neck and total hip T-scores between −2.5 and −1.0. However, spinal osteoporosis was not an exclusion criterion in the trial. Clinical trials of alendronate in osteopenia increased BMD but failed to reduce fracture risk, while a trial of combined estrogen and progestin increased BMD and reduced fracture risk but was stopped early because of increased risks of cardiovascular disease and breast cancer.^9^^,^^10^ In osteopenia, the absolute risk of fracture is lower than in osteoporosis, thus potentially diminishing the efficacy of pharmacological interventions. It is therefore important to consider the overall risk–benefit profile, including the potential side effects and complications of drug therapy.

Raloxifene has been shown to increase lumbar spine BMD and reduce fracture risk in patients with osteoporosis, with a recent study demonstrating additional BMD improvement when it is given in combination with cholecalciferol.^11^^,^^12^ Furthermore, the drug may be more widely prescribed in clinical practice given its superior safety profile when compared with bisphosphonates, particularly in the context of medication-related osteonecrosis of the jaw and atypical femoral fracture.^13^^,^^14^ Raloxifene has an advantage over combined estrogen and progestin therapy in reducing the risk of breast cancer.^15^ However, its effect on maintaining or increasing BMD in osteopenia as defined by the World Health Organization (WHO), which is defined by the lowest BMD at the lumbar spine, femoral neck, and hip, remains unexplored.^15^^,^^16^ The current trial assessed the effect of combined raloxifene and cholecalciferol on BMD in postmenopausal women with osteopenia.

## Materials and methods

### Study design

**Figure 1:**
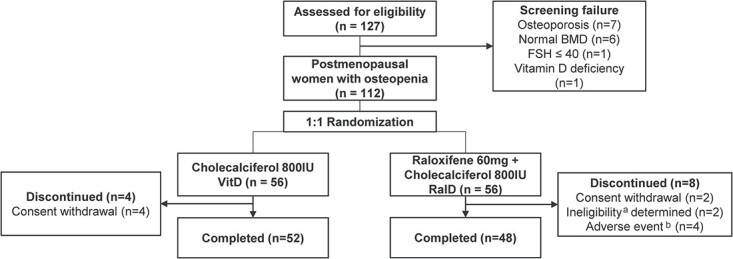
Diagram of study design. ^a^Ineligibility (1 wrist fracture, 1 requirement for active cancer treatment). ^b^Adverse events (2 leg cramps, 1 dry mouth, 1 hot flush). Abbreviations: BMD, bone mineral density; FSH, follicle-stimulating hormone.

This investigator-initiated, randomized, open-label, prospective, single-center study was conducted at Severance Hospital, Yonsei University, Seoul, Republic of Korea. The participants were randomized 1:1 into two groups: raloxifene plus cholecalciferol (RalD) or cholecalciferol alone (VitD) (Fig. 1).

The study was registered with ClinicalTrials.gov (registration number: NCT05386784). The Yonsei University College of Medicine Ethics Committee approved this study (4-2020-0977), which adhered to the ethical principles of the 1964 Declaration of Helsinki and its subsequent amendments. Written informed consent was obtained from all participants before enrolment, including protocol screening procedures and study drug administration.

### Participants

This study included postmenopausal women with osteopenia. Postmenopausal status was defined as the absence of menstruation for more than 48 wk prior to screening, with no other physiological or pathological causes of amenorrhea. In case of doubt, serum follicle-stimulating hormone (FSH) was measured for confirmation (menopause defined by FSH ≥ 40 mIU/mL). Osteopenia was defined as the lowest BMD T-score between −2.5 and −1.0 among the lumbar spine, femoral neck, and total hip, according to the WHO definition.^16^^,^^17^ We excluded participants with secondary osteoporosis (due to factors such as systemic glucocorticoid or aromatase inhibitor use, thyrotoxicosis, or hyperparathyroidism), vitamin D deficiency at baseline (25-hydroxyvitamin D (25-OHD) level < 10 ng/mL), ongoing treatment for cancer, history of vascular thrombosis, bisphosphonate treatment within the previous 12 mo, and contraindications to raloxifene use according to the summary of product characteristics. The inclusion and exclusion criteria are summarized in Table S1.

### Interventions

All participants underwent a baseline assessment, including medical history and laboratory tests, before undergoing randomization. At baseline, participants were screened for eligibility by physicians and a clinical research nurse. Subsequently, they were randomized in a 1:1 ratio to receive treatment with RalD or VitD. A random allocation sequence was computer-generated elsewhere. Participants were evaluated for tolerability and safety of the study medication at 24 and 48 wk after baseline. For the prescription, in the RalD group, raloxifene plus cholecalciferol was prescribed in a fixed-dose combination product (RaboneD®; Hanmi Pharmaceutical Co., Ltd, Seoul, Korea). RaboneD is a capsule containing raloxifene 60 mg and cholecalciferol 800 IU. RalD participants were asked to take it daily after breakfast. In the VitD group, cholecalciferol was prescribed with oral drop solution (D3base oral drops 10 000 IU/ml; ABIOGEN PHARMA S.p.A, Pisa, Italy). The VitD participants were asked to take 4 drops of D3base oral drops, equivalent to 800 IU of cholecalciferol, every day after breakfast using a dropper. Dietary calcium intake was not restricted and additional calcium supplements were not supplied. The 6-mo prescription was initially provided to the patient and was subsequently re-prescribed at each 24-wk visit to the outpatient office. The study was stopped if participants met any of the following criteria: protocol deviation, ineligibility, patient request, noncompliance, loss to follow-up, or investigator decision. Participants who experienced a clinical fracture during the study were considered to have progressed to osteoporosis, and their participation in the study was discontinued. The risk of further fractures and alternative treatment options were discussed with the investigator with the affected participants.

Funding and trial medication were provided by Hanmi Pharmaceutical Co., Ltd. The funding agencies had no direct role in the study design, data collection, analysis, interpretation, or writing of this report.

### BMD measurements and spine radiography

BMD was measured by DXA (Discovery W, Hologic Inc.; NH, USA) at baseline and follow-up (48 wk after the first treatment) in the lumbar spine, femoral neck, and total hip. BMD T-scores were calculated using the manufacturer’s reference range. Changes in BMD are expressed as mean ± standard deviation (SD) with percentage changes. Routine quality control of the bone density equipment according to the manufacturer’s protocol was performed at our site. The least significant change (LSC), calculated at the 95% confidence level of the coefficient of variation (CV), were 2.78%, 6.52%, and 3.27% for the lumbar spine, femoral neck, and total hip, respectively. The 10-yr probability of major osteoporotic fracture (MOF) and hip fracture (HF) was calculated using a computer-based algorithm (http://www.shef.ac.uk/FRAX). Vertebral morphometry was assessed using spine radiography at baseline and 48 wk after treatment.

### Covariates

Height and weight were measured using an automated digital stadiometer and weighing scale. At baseline, participants were asked about their previous fracture history, smoking history, and alcohol consumption. Participants were defined as exercising if they regularly exercised at least twice a wk for at least 30 min.

Blood samples were collected after an overnight fast of at least 8 h. White blood cell, serum hemoglobin, total platelet counts, glucose, aspartate aminotransferase, alanine aminotransferase, total cholesterol, triglyceride, and high-density lipoprotein cholesterol concentrations were measured using an ADVIA 1650 chemistry system (Siemens Medical Solutions, Tarrytown, NY, USA). Serum creatinine, calcium, and phosphate concentrations were measured using a Hitachi chemistry autoanalyzer 7600-110 (Hitachi Ltd., Tokyo, Japan). Serum procollagen type 1 N-terminal propeptide (P1NP; Roche Diagnostics; intra-assay CV < 3.6%, interassay CV < 3.9%) and C-terminal telopeptide (CTX; Elecsys β-CrossLaps; Roche Diagnostics, Mannheim, Germany; intra-assay CV < 3.5%, interassay CV < 8.4%) were measured, and serum 25-OHD levels were measured using a radioimmunoassay (RIA; DiaSorin, Inc.; Stillwater, MN, USA; intra-assay CV < 4.1%, interassay CV < 7.0%). At each visit, participants had a blood sample taken for testing and all tests were carried out in the central laboratory at Severance Hospital.

### Outcomes

The primary outcome was the mean percent change in lumbar spine BMD from baseline to follow-up. The secondary outcomes were mean percent changes in BMD in the femoral neck and total hip. A good responder was defined as a BMD change greater than the LSC and the proportion was compared between the groups. Bone turnover markers (BTM) were measured at 24-wk intervals and the change over time in both groups was another secondary outcome. Occurrences of symptomatic, morphometric vertebral fracture, and other fragility fractures were assessed using spine radiography at baseline and after 48 wk of trial.

### Statistical analysis

This study was designed to have 90% power (two-sided, α = .05) to detect the superiority of raloxifene plus cholecalciferol versus cholecalciferol alone, using Power Analysis & Sample size (PASS version 2008; NCSS Statistical Software, Kaysville, UT, USA). The study sample size was 56 participants per treatment group to detect a mean difference in lumbar spine BMD change of 3.2%, assuming an SD of 4.4%, with an expected dropout rate of 10%.

Baseline characteristics are presented as mean ± SD or median (interquartile range) for continuous variables, and as frequencies (%) for categorical variables. Study participants were compared using independent two-sample *t*-tests, Wilcoxon rank-sum tests, and χ2 tests, as appropriate. Changes in mean percent (%) and absolute value of BMD from baseline to 48 wk of treatment were compared between the RalD and VitD groups using an independent two-sample *t*-test. BMD changes are presented as the mean + standard error of the mean (SEM). The independent effect of raloxifene on BMD in the lumbar spine and total hip was analyzed using multiple linear regression analysis. The differences in the proportion of good responders (BMD increase ≥ LSC) were evaluated using the χ^2^ test. Further subgroup analyses were performed with interaction terms according to the degree of osteopenia based on the lowest BMD T-score of the lumbar spine, femoral neck, and total hip T-scores and baseline 25-OHD levels (<30 or ≥30 ng/mL). The severity of osteopenia was classified based on studies that had previously assessed the rate of progression to osteoporosis in osteopenia.^18^ Those with the lowest T-score between −2.0 and − 1.0 were assigned to the mild-to-moderate osteopenia group, and those with the lowest T-score of −2.0 or less were assigned to the severe osteopenia group. Differences in BTM were analyzed using a linear mixed model. All statistical analyses were performed using Stata 16.1 (Stata Corp., College Station, TX, USA). Graphics were produced using GraphPad Prism (version 8.0.2; GraphPad Software Inc., San Diego, CA, USA). The statistical significance level was set at a two-sided *P*-value <.05.

## Results

### Baseline characteristics of study participants

From December 2020 to June 2021, 127 participants were assessed for eligibility, 112 of them were randomly assigned to the RalD (*n* = 56) or VitD (*n* = 56) group. Of the participants, 48 (86%) in the RalD group and 52 (93%) in the VitD group completed the study. The study design and reasons for discontinuation are described in Fig. 1.

At baseline, the mean age was 63.1 ± 6.8 yr, and the prevalence of a vertebral fracture history, smoking, alcohol consumption, and the degree of physical activity was similar between the two groups. However, body mass index (BMI) and BMD T-score in the lumbar spine (RalD vs. VitD: −1.76 vs. −1.46), femoral neck (−1.74 vs. −1.50), and total hip (−0.81 vs. −0.44) were lower, and 25-OHD level was higher in the RalD group compared with VitD group. FRAX score, the 10-yr probability of MOF and HF, was not significantly different between groups (Table 1). The baseline characteristics of those who completed the 48 wk of the study are described separately in Table S2.

**Table 1:** Baseline characteristics according to study groups.

	VitD (*n* = 56)	RalD (*n* = 56)	*P*-value
Age (years), mean ± SD	63.1 ± 7.1	63.2 ± 6.6	.967
BMI (kg/m^2^), mean ± SD	24.2 ± 3.1	23.0 ± 2.6	.024
Smoking, *n* (%)	0 (0%)	0 (0%)	1.00
Consumes alcohol, *n* (%)	4 (7%)	3 (5%)	.696
Physical activity, *n* (%)	34 (61%)	37 (66%)	.556
Lumbar spine T-score, mean ± SD	−1.46 ± 0.63	−1.76 ± 0.47	.006
Femoral neck T-score, mean ± SD	−1.50 ± 0.68	−1.74 ± 0.48	.035
Total hip T-score, mean ± SD	−0.44 ± 0.74	−0.81 ± 0.48	.002
CTX (ng/mL), median (IQR)	0.48 (0.28–0.69)	0.47 (0.38–0.66)	.629
P1NP (ng/mL), median (IQR)	49.6 (36.5–57.9)	50.0 (43.6–63.2)	.330
25-OHD (ng/mL), mean ± SD	30.0 ± 14.7	36.0 ± 13.2	.024
FRAX-MOF (%), mean ± SD	6.4 ± 2.1	6.9 ± 2.2	.206
FRAX-HF (%), mean ± SD	1.7 ± 1.1	2.0 ± 1.1	.108

Abbreviations: 25-OHD, 25-hydroxyvitamin D; BMI, body mass index; CTX, C-terminal telopeptide; FRAX, Fracture Risk Assessment Tool; HF, hip fracture; IQR, interquartile range; MOF, major osteoporotic fracture; P1NP, procollagen type 1 N-terminal propeptide; RalD, raloxifene plus cholecalciferol; SD, standard deviation; VitD, cholecalciferol alone.

**Table 2:** Mean percent and absolute change in BMD from baseline to 48 weeks after the first treatment (RalD vs. VitD).

	Mean (SEM)	95%CI (*P*-value)
BMD mean percent change by DXA		
BMD lumbar spine (%)	3.2 (1.1)	1.0–5.4 (0.004)
BMD femoral neck (%)	2.2 (0.9)	0.4–4.1 (0.020)
BMD total hip (%)	2.6 (0.9)	0.9–4.3 (0.003)
BMD absolute change by DXA		
BMD lumbar spine (g/cm^2^)	0.024 (0.009)	0.006–0.041 (0.008)
Lumbar spine T-score	0.19 (0.08)	0.04–0.34 (0.013)
BMD femoral neck (g/cm^2^)	0.013 (0.006)	0.002–0.025 (0.024)
Femoral neck T-score	0.14 (0.06)	0.03–0.25 (0.015)
BMD total hip (g/cm^2^)	0.019 (0.006)	0.006–0.033 (0.003)
Total hip T-score	0.17 (0.05)	0.06–0.28 (0.003)

Data are mean (SEM) unless otherwise stated, and 95% CIs and *P*-values are nominal.

Abbreviations: BMD, Bone mineral density; DXA, Dual X-ray absorptiometry; RalD, raloxifene plus cholecalciferol; VitD, cholecalciferol alone.

### Effects of raloxifene on BMD in postmenopausal women with osteopenia

**Figure 2:**
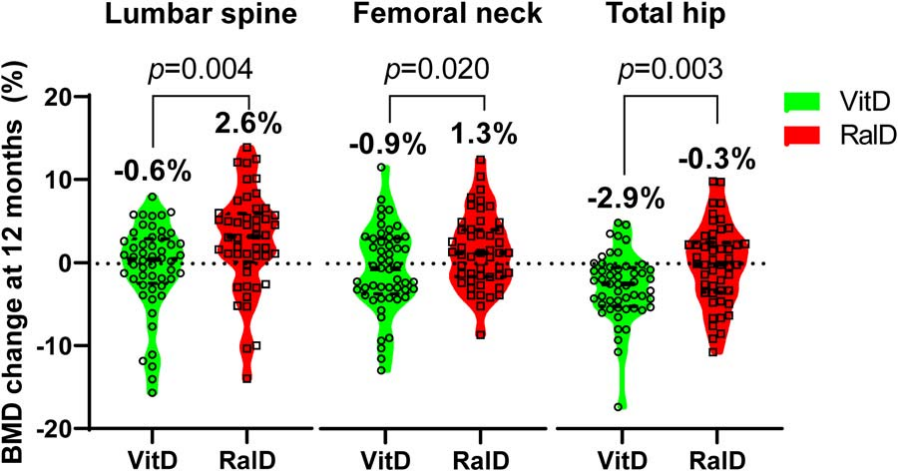
BMD changes with VitD or RalD in postmenopausal women with osteopenia. The dashed line indicates the median and the dotted line indicates the interquartile range. Abbreviations: RalD, raloxifene plus cholecalciferol; VitD, cholecalciferol alone.

From baseline to 48 wk after treatment, the RalD group showed greater lumbar spine BMD gain than the VitD group (RalD vs. VitD; +2.6% vs. −0.6%, *P* = .004). In the femoral neck, the RalD group showed significant BMD gain in the femoral neck (RalD vs. VitD; +1.3 vs. −0.9, *P* = .020) and significantly less bone loss in the total hip (RalD vs. VitD; −0.2% vs. −2.8%, *P* = .003) (Table 2) (Fig. 2). As there was a difference in BMD at baseline, further absolute BMD change and regression analysis were performed. When comparing absolute BMD change between the two groups, there were also significant differences at all three sites (RalD vs. VitD; lumbar spine +0.019 g/cm^2^ vs. −0.004 g/cm^2,^  *P* = .008; femoral neck +0.007 g/cm^2^ vs. −0.006 g/cm^2^, *P* = .024; total hip −0.003 g/cm^2^ vs. −0.023 g/cm^2^, *P* = .003). In regression analysis, the effect of raloxifene in lumbar spine BMD remained significant in the multiple linear regression model (adjusted β +3.05% vs. VitD, *P* = .015) after adjusting for age, BMI, baseline lumbar spine BMD T-score, and 25-OHD level (Table 3).

**Figure 3:**
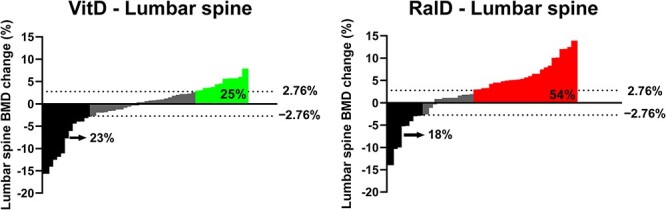
Waterfall plot of lumbar spine bone mineral density changes over time. Favorable responses were defined as those with a BMD increase of more than the LSC ≥ 2.76% in the lumbar spine. Abbreviations: BMD, bone mineral density; RalD, raloxifene plus cholecalciferol; VitD, cholecalciferol alone.

When an increase in BMD above LSC was defined as a good response, significantly more participants in the RalD group had increased lumbar spine BMD above LSC (2.76%) compared with the VitD group (RalD vs. VitD; 54% vs. 25%, *P* = .003) (Fig. 3). No new morphometric or clinical vertebral fractures were reported in either group during the 48 wk of treatment. One participant (2%) in the RalD group experienced a wrist fracture at week 47 of the study and was withdrawn from the study following the study protocol and reassessed for fracture risk for further treatment.

### Changes in bone turnover markers and 25-OHD

**Figure 4:**
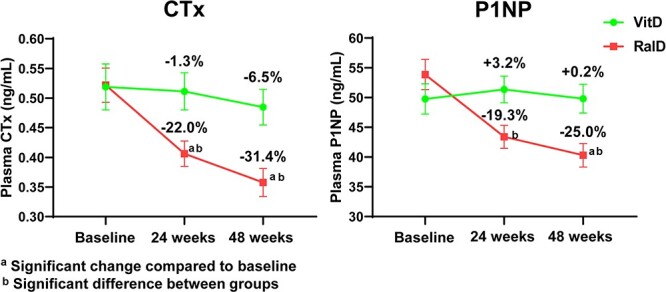
Percent changes in bone turnover markers. ^a^Significant change compared to baseline, ^b^Significant difference between groups. Abbreviations: CTX, C-terminal telopeptide; P1NP, procollagen type 1 N-terminal propeptide; RalD, raloxifene plus cholecalciferol; VitD, cholecalciferol alone.

The percentage changes in serum P1NP and CTX compared with baseline at each time point are shown in Fig. 4. In the RalD group, P1NP was significantly lower at 24 (−16.2%) and 48 wk (−21.4%), and CTX was significantly lower at 48 wk (−23.4%) compared with baseline. In the VitD group, neither P1NP nor CTX showed significant differences compared with baseline. Concentrations of P1NP and CTX were significantly lower in the RalD group compared with the VitD group at 24 and 48 wk, and 25-OHD levels were 26.9 ng/mL in the RalD group and 26.6 ng/mL in the VitD group at week 48, with no significant difference between the two groups (*P* = .855).

**Table 3:** Effect of raloxifene plus cholecalciferol on lumbar spine BMD compared to cholecalciferol monotherapy.

Variables	Unadjusted		Adjusted	
	Unadjusted β (95% CI)	*P-*value	Adjusted β (95% CI)	*P-*value
RalD (vs. VitD)	3.23 (1.03–5.44)	.005	3.05 (0.62–5.49)	.015
Age (per 1 year older)	0.03 (−0.14 to 0.19)	.330	0.02 (−0.15 to 0.19)	.804
Body mass index (per 1 kg/m^2^ lower)	0.14 (−0.25 to 0.53)	.464	0.00 (−0.44 to 0.43)	.983
Lumbar spine T-score at baseline (per 1 unit lower)	1.05 (−0.93 to 3.02)	.296	−0.04 (−2.26 to 2.18)	.973
25-OHD (per 1 ng/mL lower)	−0.05 (−0.13 to 0.03)	.206	0.03 (−0.06 to 0.11)	.538

Abbreviations: 25-OHD, 25-hydroxy-vitamin D; CI, confidence interval; RalD, raloxifene plus cholecalciferol; VitD, cholecalciferol alone.

### Subgroup analysis with the severity of osteopenia and 25-OHD

**Figure 5:**
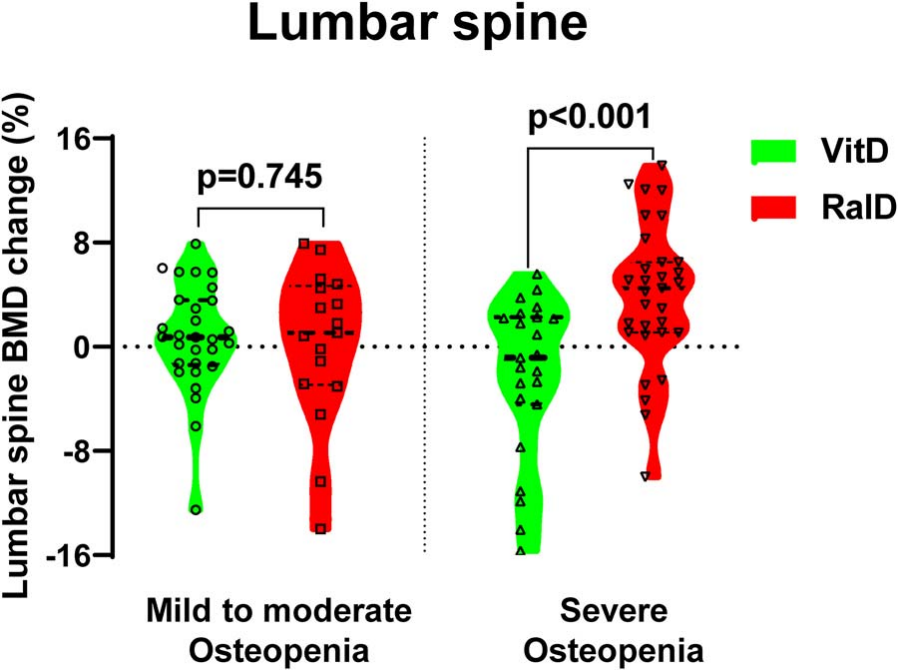
Lumbar spine BMD change according to baseline lowest T score. The dashed line indicates the median and the dotted line indicates the interquartile range. Mild to moderate osteopenia: −2.0 < lowest T-score < −1.0. Severe osteopenia: −2.5 < lowest T-score ≤ −2.0. Abbreviations: BMD, bone mineral density; RalD, raloxifene plus cholecalciferol; VitD, cholecalciferol alone.

In subgroup analysis, participants were divided into two groups based on their lowest BMD T-score and 25-OHD levels. In participants with mild to moderate osteopenia (−2.0 < lowest T-score < −1.0 at baseline), raloxifene showed no significant difference between the two groups at the lumbar spine (RalD vs. VitD; +0.2% vs. +0.7%, *P* = .745). However, in severe osteopenia (−2.5 < lowest T-score ≤ −2.0 at baseline), the RalD group showed significant BMD gain in the lumbar spine compared with the VitD group (RalD vs. VitD; +4.0% vs. −2.2%, *P* < .001) (Fig. 5). These differences were still robust in subgroup analysis with significant *P* for interaction (adjusted β 6.3%; 95% confidence interval 2.7–9.9; *P* for interaction = .002) (Table 4). Further analysis of baseline 25-OHD divided by 30 ng/mL showed a more robust result in the RalD group than in the VitD group, with no significant *P*-for interaction between the two subgroups.

**Table 4:** Subgroup analysis of lumbar spine BMD change (RalD vs. VitD) according to the lowest T-score and 25-OHD at baseline.

Variables	Number of participants	Adjusted β (95% CI)	*P*-value	*P* for Interaction
Osteopenia				
Mild to moderate	RalD *n* = 17 VitD *n* = 29	−0.8 (−4.1 to 2.4)	.599	Ref
Severe	RalD *n* = 31 VitD *n* = 23	+6.3 (2.7–9.9)	.001	.002
25-OHD				
<30 ng/mL	RalD *n* = 19 VitD *n* = 31	3.0 (0.2–5.9)	.037	Ref
≥30 ng/mL	RalD *n* = 29 VitD *n* = 21	3.2 (−0.4 to 6.7)	.079	.092

Adjusted for age, body mass index, baseline lumbar spine BMD, and 25-OHD.

Mild to moderate osteopenia: −2.0 < Lowest T-score < −1.0.

Severe osteopenia: −2.5 < Lowest T-score ≤ −2.0.

Abbreviations: 25-OHD, 25-hydroxy-vitamin D; RalD, raloxifene plus cholecalciferol; VitD, cholecalciferol alone.

## Discussion

In postmenopausal women with osteopenia, raloxifene plus cholecalciferol for 48 wk improved lumbar spine BMD attenuated total hip BMD loss compared with taking cholecalciferol alone. These results were similar for absolute BMD change and differences in lumbar spine BMD remained significant in the multiple regression model after adjustment for covariates. In subgroup analysis, the difference in lumbar spine BMD gain between the two groups was robust in those with severe osteopenia group.

Several previous studies have shown the effectiveness of raloxifene in postmenopausal women without osteoporosis. In a phase 2 trial involving 152 postmenopausal women with a lumbar spine BMD T-score between −2.5 and +2.0, raloxifene showed BMD gains of 2.4% and 2.0% at the lumbar spine and total hip, respectively.^19^ In another trial with healthy postmenopausal women, raloxifene increased both lumbar spine and total hip BMD by 1.1% compared with baseline and showed a more pronounced increase in BMD compared with that of the placebo.^20^ The re-analysis of the Multiple Outcomes of Raloxifene Evaluation (MORE) trial,^21^ in which osteopenia was defined by total hip BMD, showed that raloxifene increased BMD and also reduced the risk of new morphological and clinical fractures by 47% and 75%, respectively, compared with those of the placebo. The incidence of new vertebral fractures in women treated with raloxifene was similar in those with osteoporosis (2%) and osteopenia (1.9%). However, the MORE study did not meet the WHO definition of osteopenia, as it initially recruited patients who had a T-score of −2.5 or less in the lumbar spine or femoral neck, or a radiographically apparent vertebral fracture. Our study showed similar results to previous studies in terms of BMD gains but differed in that it recruited participants with the lowest BMD T-scores between −2.5 and −1.0 among the lumbar spine, femoral neck, and total hip. Further large, long-term follow-up studies are warranted to establish the effect of raloxifene on fracture risk reduction in osteopenia.

Fracture prevention in osteopenia should be based on an individualized assessment of fracture risk, and the decision to administer a drug should balance the cost-effectiveness against the harms of the drug.^22^ Previous studies evaluating the effectiveness of oral alendronate in osteopenia have not demonstrated cost-effectiveness.^23^^,^^24^ The cost-effectiveness of preventive therapy of raloxifene was compared in one study with alendronate in postmenopausal osteopenia.^25^ The study found that in 55- to 60-yr-old women, treatment with raloxifene was more cost-effective than alendronate. A recent study from Korea^26^ showed that treatment with raloxifene was more cost-effective than calcium and vitamin D alone for the prevention of fragility fractures in patients with femoral neck T-scores near the threshold for osteoporosis between −2.4 and −2.0. Osteopenia covers a wide range of T-scores between −2.5 and −1.0, which, when divided by 0.5, shows different risks of progression to osteoporosis or fracture.^18^^,^^27^ Studies that have analyzed the appropriate interval for BMD testing have recommended that individuals with severe osteopenia, with a T-score between −2.5 and −2.0, should test their BMD every year, given that ~10% of osteopenia cases may progress to osteoporosis, or rarely a fracture before osteoporosis treatment. In our study, raloxifene increased lumbar spine BMD and showed even more robust results in severe osteopenia. Our results provide proof-of-concept level evidence to support the strategy of combining raloxifene with cholecalciferol as a treatment option in postmenopausal women with osteopenia, particularly those with the lowest T score of −2.0 or less. However, this hypothesis needs further validation in larger fracture risk reduction and cost-effectiveness trials.

The strengths of our study are defining osteopenia according to the WHO definition by the inclusion of lumbar spine T-score and the inclusion of a much younger age group compared with that of previous studies. We also showed that the effect of BMD gain differed significantly according to the severity of osteopenia, which may help clinicians’ decision-making in a real-world setting. However, our study also has several limitations. First, it was conducted as an open-label trial and prescribed different formulations to supplement cholecalciferol. This might cause additional biases and overestimate adverse events. However, given the unavailability of a pill formulation containing 800 IU of cholecalciferol alone in Korea, we used an oral drop solution of vitamin D as a control and found no difference in the 25-OHD levels between the two groups at 48 wk. Second, this study had limited power to assess fracture prevention due to the relatively small number of participants and the short follow-up period. Third, BMD and serum 25-OHD levels were different between the two groups at baseline, indicating an imbalance between the two groups despite randomization. As our primary outcome was the percentage change in BMD in the lumbar spine, the differences between the two groups should be interpreted with caution. However, we attempted to overcome this limitation by further analyzing the absolute change in BMD and using multiple linear regression. The absolute BMD changes were still significantly higher in the RalD group, and multiple linear regressions in the lumbar spine showed similar results even after adjustment for several variables, including baseline BMD and 25-OHD levels. Our study results provide an impetus for future randomized controlled trials in postmenopausal women with osteopenia, particularly those close to osteoporosis.

In conclusion, raloxifene plus cholecalciferol in postmenopausal women with osteopenia improved lumbar spine BMD and attenuated total hip BMD loss compared with conservative treatment with cholecalciferol alone. These results suggest that raloxifene plus cholecalciferol could be an effective treatment option for postmenopausal women with osteopenia, particularly those with the lowest T score of −2.0 or less. Further studies with larger sample sizes and longer follow-up periods are needed to determine whether this intervention can be generalized to clinical practice.

## Supplementary Material

Supplementary_Information_ziae073

## Data Availability

The datasets used and analyzed during the study are available from the corresponding author on reasonable request.
