# Supplementary material for: A randomized controlled trial of the effect of raloxifene plus cholecalciferol versus cholecalciferol alone on bone mineral density in postmenopausal women with osteopenia
Source: JBMR Plus. 2024 May 30;8(7):ziae073. doi: 10.1093/jbmrpl/ziae073 (PMC11208723; doi:10.1093/jbmrpl/ziae073)
Supplement: Supplementary_Information_ziae073 [file supplementary_information_ziae073.docx]

**A randomized controlled trial of the effect of raloxifene plus cholecalciferol versus cholecalciferol alone on bone mineral density in postmenopausal women with osteopenia**

Sungjae Shin^1*^, Namki Hong^2*^, Yumie Rhee^2^

^1^ Division of Endocrinology and Metabolism, Department of Internal Medicine, National Health Insurance Service Ilsan Hospital, Goyang, Korea

^2^ Department of Internal Medicine, Endocrine Research Institute, Yonsei University College of Medicine, Seoul, 03722 Republic of Korea

^*^These authors contributed equally to this work.

**Corresponding author**

Yumie Rhee, M.D., Ph.D.

Department of Internal Medicine, Endocrine Research Institute, Yonsei University College of Medicine 50‑1 Yonsei‑ro, Seodaemun‑gu, Seoul 03722, Republic of Korea

Tel.: (+82)-2-2228-1973, Fax: (+82)-2-392-5548

Email: [YUMIE@yuhs.ac](mailto:YUMIE@yuhs.ac)

ORCID: http://orcid.org/0000-0003-4227-5638

**Supplementary Table 1** Inclusion and exclusion criteria of study participants

| **Inclusion criteria** | **Exclusion criteria** |
| --- | --- |
| 1. Postmenopausal women defined as no menstruation for more than 48 weeks prior to screening and no other pathological or physiological causes. If in doubt, a serum follicle-stimulating hormone (FSH) test may be performed at screening) 2. Osteopenia ( −2.5 < T-score < −1.0 by DXA) | 1. Secondary osteoporosis (Systemic glucocorticoid use, aromatase inhibitor administration, thyrotoxicosis, hyperparathyroidism, etc.) 2. Vitamin D deficiency at baseline (25-OHD < 10 ng/mL) 3. Active cancer treatment 4. History of vascular thrombosis 5. Bisphosphonate treatment within the last 12 months 6. Contraindication for raloxifene according to the summary of product characteristics |

25-OHD, 25-hydroxyvitamin D; DXA, dual-energy X-ray absorptiometry.

**Supplementary Table 2** Baseline characteristics of the study participants who completed the trial according to group.

|  | **VitD (n = 52)** | **RalD (n = 48)** | ***p* Value** |
| --- | --- | --- | --- |
| Age (years), mean ± SD | 63.1 ± 7.3 | 63.0 ± 6.6 | 0.956 |
| BMI (kg/m^2^), mean ± SD | 24.3 ± 3.2 | 23.0 ± 2.6 | 0.041 |
| Smoking, n (%) | 0 (0%) | 0 (0%) | 1.00 |
| Consumes alcohol, n (%) | 4 (8%) | 2 (4%) | 0.458 |
| Physical activity, n (%) | 31 (60%) | 33 (69%) | 0.342 |
| Lumbar spine T-score, mean ± SD | −1.42 ± 0.62 | −1.82 ± 0.46 | <0.001 |
| Femoral neck T-score, mean ± SD | −1.49 ± 0.65 | −1.73 ± 0.47 | 0.038 |
| Total hip T-score, mean ± SD | −0.45 ± 0.74 | −0.80 ± 0.50 | 0.007 |
| CTX (ng/mL), median (IQR) | 0.47 (0.27 to 0.69) | 0.47 (0.37 to 0.67) | 0.644 |
| P1NP (ng/mL), median (IQR) | 50.0 (37.3 to 58.9) | 49.8 (44.1 to 63.2) | 0.377 |
| 25-OHD (ng/mL), mean ± SD | 30.0 ± 15.2 | 36.1 ± 13.4 | 0.036 |
| FRAX − MOF (%), mean ± SD | 6.4 ± 2.1 | 6.9 ± 2.3 | 0.196 |
| FRAX − HF (%), mean ± SD | 1.6 ± 1.1 | 2.0 ± 1.1 | 0.102 |

VitD, cholecalciferol alone; RalD, raloxifene plus cholecalciferol; SD, standard deviation; BMI, body mass index; VFx, vertebral fracture; CTX, C-terminal telopeptide; P1NP, procollagen type 1N-terminal propeptide; 25-OHD, 25-hydroxyvitamin D; FRAX, Fracture Risk Assessment Tool; MOF, major osteoporotic fracture; HF, hip fracture; IQR, interquartile range
